# Microtubule Hyperacetylation Enhances KL1-Dependent Micronucleation under a Tau Deficiency in Mammary Epithelial Cells

**DOI:** 10.3390/ijms19092488

**Published:** 2018-08-23

**Authors:** Haruka Sudo

**Affiliations:** 1 Faculty of Health Science, Tokoha University, 1-30, Mizuochi-cho, Aoi-ku, Shizuoka-shi, Shizuoka 420-0831, Japan; sudoh@sz.tokoha-u.ac.jp or sudoh@tky.ndu.ac.jp; Tel.: +81-(54)-297-3234; 2 Department of Biochemistry, The Nippon Dental University School of Life Dentistry at Tokyo, 1-9-20 Fujimi, Chiyoda-ku, Tokyo 102-8159, Japan; 3 Department of Anatomy, Keio University School of Medicine, 35 Shinanomachi, Shinjuku-ku, Tokyo 160-8582, Japan

**Keywords:** breast cancer, microtubule acetylation, microtubule severing, mitotic spindle, tau

## Abstract

Enhanced microtubule acetylation has been identified as a negative prognostic indicator in breast cancer. We reported previously that primary cultured human mammary epithelial cells manifest breast cancer-related aneuploidization via the activation of severing protein katanin-like (KL)1 when tau is deficient. To address in this current study whether microtubule hyperacetylation is involved in breast carcinogenesis through mitosis, the effects of tubacin on human mammary epithelial cells were tested using immunofluorescence techniques. Tau-knockdown cells showed enhancement of KL1-dependent events, chromosome-bridging and micronucleation in response to tubacin. These enhancements were suppressed by further expression of an acetylation-deficient tubulin mutant. Consistently, using a rat fibroblast-based microtubule sensitivity test, it was confirmed that KL1 also shows enhanced activity in response to microtubule hyperacetylation as well as katanin. It was further observed in rat fibroblasts that exogenously expressed KL1 results in more micronucleation under microtubule hyperacetylation conditions. These data suggest that microtubule acetylation upregulates KL1 and induces more aneuploidy if tau is deficient. It is thus plausible that microtubule hyperacetylation promotes tumor progression by enhancing microtubule sensitivity to KL1, thereby disrupting spindle microtubules and this process could be reversed by the microtubule-binding and microtubule protective octapeptide NAPVSIPQ (NAP) which recruits tau to the microtubules.

## 1. Introduction

Acetylation is one of the posttranslational modifications of microtubules. It occurs on α-tubulin and is unique among the various microtubule modification processes in that it occurs on the luminal face of the microtubule (for review, see [1,2,3]). Microtubule acetylation occurs on lysine 40 (K40), which is embedded within the tubulin subunit. Two different enzymes have been identified that can remove this acetyl group, namely histone deacetylase (HDAC)6 and sirtuin (Sirt)2. The acetylation of the K40 residue of α-tubulin is principally carried out by the recently identified α-tubulin *N*-acetyltransferase (α-TAT)1, which uses acetyl-CoA as the source of the acetyl moiety [4]. K40 acetylation has so far been linked to cell migration [5,6], touch sensation [7], intracellular trafficking [8,9], and cell adhesion [10].

Microtubules are highly acetylated in mitotic cells [11,12]. During metaphase, acetylated tubulin is enriched at the interpolar and kinetochore microtubules but not at the astral microtubules. In addition, acetylated tubulin becomes concentrated at the midbody during telophase and cytokinesis [12,13]. However, the physiological function of this modification in mitosis is still unclear. There are cell lines such as PtK2 which do not show microtubule acetylation [4,12] and some reports claim that microtubule acetylation has no prominent function during mitosis [14]. However, recent studies have also indirectly suggested that microtubule acetylation has a normal function during mitosis and that mitotic progression is disrupted if it drops below this normal threshold [15]. Other reports have argued that the mitotic spindle functions are impaired by excessive microtubule acetylation [16]. The functional aspects of microtubule acetylation thus remain to be fully elucidated, particularly for mitosis.

Higher levels of microtubule acetylation have been observed in head-neck, pancreatic, and breast cancers [17,18,19]. In breast cancer, enhanced acetylation of microtubule has been linked to basal cell subtypes that have a poorer prognosis [19]. In that study also, the enhanced microtubule acetylation was found to promote metastatic activity.

Our previous study has suggested that KL1, one of the microtubule severing proteins, is abnormally up-regulated in early breast carcinogenesis when tau is decreased and contributes to tumor progression by inducing abnormal karyotypes that are relevant to the basal cell subtype [20]. Regarding the perturbation of the regulatory mechanisms underlying microtubule severing, we have also reported that microtubule hyperacetylation enhances the activity of katanin but not spastin [21]. Notably, human KL1 has a higher homology with katanin than with spastin. For example, human KL1 has an 85% identity with human katanin in the conserved AAA ATPase domain [22], but only a 48% identity with human spastin in this domain [23]. Based on this prior evidence, a plausible hypothesis was that human KL1 would also be sensitive to the acetylation status of the substrate microtubules.

The pathological implications of enhanced microtubule acetylation during breast cancer progression are explored in this present study, with a focus on mitosis. Using rat fibroblasts, a microtubule severing sensitivity test was first performed under hyperacetylation conditions. The mitotic spindle and micronucleation were analyzed in response to microtubule hyperacetylation in the same cells. The micronucleation/microtubule acetylation relationship was further explored in normal human mammary epithelial cells (HMECs), which express endogenous tau, and in which the loss of tau has been suggested to promote early stage breast carcinogenesis [20]. It was observed that only under an imbalance between tau and KL1 did increased microtubule acetylation enhance abnormal chromosome segregation and micronucleation.

## 2. Results

### 2.1. Microtubules Rich in Acetylated Tubulin Are Preferentially Severed by KL1

The rat fibroblast (RFL)-6, a cell line previously employed in studies of microtubule-severing proteins [20,21,24,25,26] was used in the present analyses. Fibroblasts are useful for high-resolution imaging of microtubules because they have a flat morphology. In addition, they do not endogenously express tau which would lower the sensitivity of the microtubules to severing [20,24,26].

**Figure 1:**
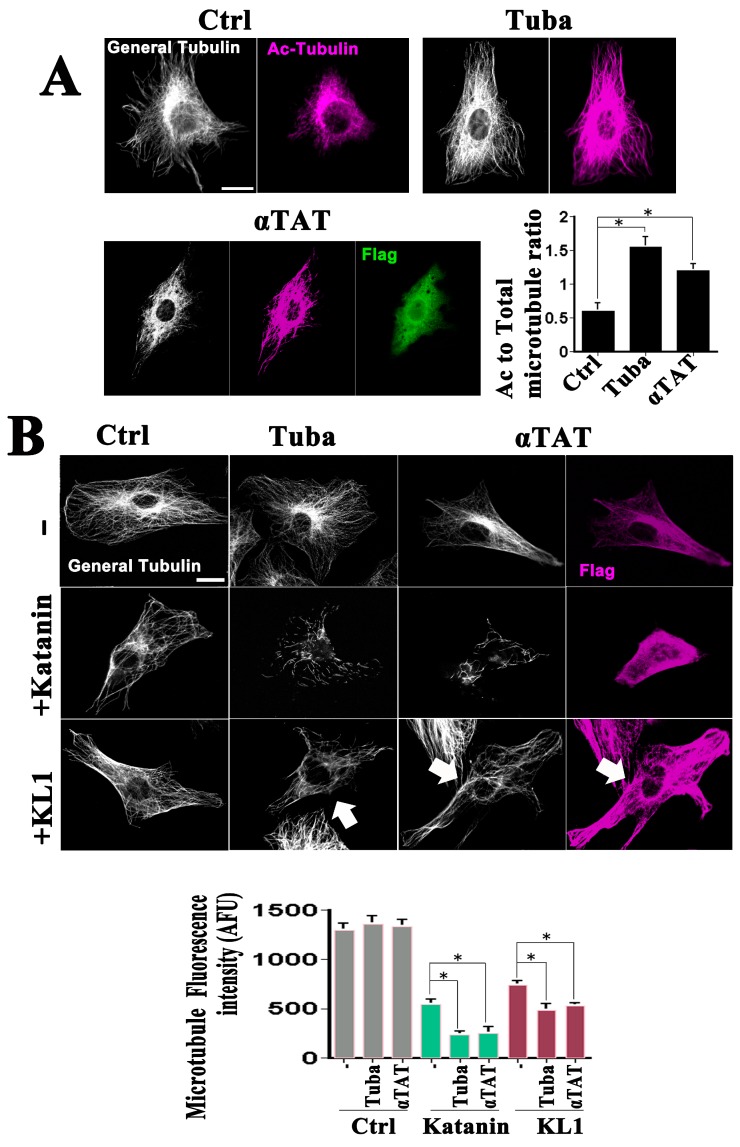
Effects of enhanced microtubule acetylation on KL1-mediated microtubule severing in RFL-6 cells. (**A**) Effects of tubacin treatment and flag-tagged α-TAT1 overexpression on cellular microtubule acetylation in RFL-6 cells. Cells were treated with tubacin or transfected with flag-tagged α-TAT1 expression plasmids, cultured, and stained using anti-tubulin, anti-acetylated-tubulin, and anti-flag antibodies. In the immunofluorescence images, the white signals indicate general tubulin (General Tubulin), magenta denotes acetylated tubulin (Ac-Tubulin), and green signals indicate the expressed flag tag (Flag). Compared with the control cells (Ctrl), both the tubacin-treated cells (Tuba) and α-TAT1 overexpressing cells (αTAT) showed enhanced microtubule acetylation. Scale bar, 10 µm. The graph shows the quantified acetylated-tubulin/total-tubulin signal ratios. Significant increases were detected between control vs. tubacin or control vs. α-TAT1. (**B**) Representative images of the severing protein sensitivity test results in RFL-6 cells. GFP-katanin (+Katanin) overexpressing cells under α-TAT1 co-expression conditions showed a similar enhancement of microtubule reduction to that observed under tubacin treatment. GFP-KL1 (+KL1) expressing cells showed a moderate reduction in microtubules. Under tubacin treatment or α-TAT1 overexpression the GFP-KL1 expressing cells also showed an enhanced microtubule reduction. Arrows indicate GFP-KL1 expressing cells. The quantification of the total microtubule levels is indicated in the graph. Compared with the control cells, both tubacin-treated and α-TAT1-overexpressing cells showed a trend towards an increase in microtubules, but this was not significant. There was a significant increase in microtubule reduction in the Katanin + α-TAT1 compared with the Katanin-expressing cells. KL1 overexpressing cells showed a significant microtubule reduction, compared with the controls. The KL1-induced microtubule reduction was significantly enhanced by both tubacin treatment and α-TAT1 overexpression. AFU, arbitrary fluorescence unit. Scale bar, 10 µm. The asterisks indicate significant differences (Student’s *t*-test, *p* < 0.01).

To investigate the effects of enhanced microtubule acetylation on microtubule severing, previously characterized tools were employed such as tubacin (10 µM), an inhibitor of the principal tubulin deacetylase in vertebrate cells, HDAC6 [21]. An increase was observed in tubulin acetylation in the tubacin-treated cells as assessed by immunofluorescence (Figure 1A, Ctrl vs. Tuba; Figure 1A graph, ratio of acetylated to total tubulin). Because acetyl transferases and deacetylases do not exclusively affect tubulin and may have effects on microtubules that are separate from their acetylation, another approach was taken to enhance acetylation. The chief tubulin acetyl transferase in vertebrate cells, α-TAT1 [4], was found to be overexpressed. Validation of the effects of α-TAT1 on increased microtubule acetylation is shown in Figure 1A (Ctrl vs. αTAT; graph). It was necessary to select the most highly overexpressing cells to visualize the notable elevation in acetylation and these cells were used in all of the subsequent experiments. No prominent morphological change in the microtubule arrays in the tubacin-treated and α-TAT1-overexpressing cells was observed (Figure 1A) which was consistent with the results of previous reports [19,21]. Quantitative analysis (see Figure 1B, graph and upper panels) revealed slight but not significant increases in the total microtubule levels in tubacin-treated and α-TAT1-overexpressing cells, respectively. Western blot analysis (Figure S1A) revealed significant increases in the ratios of acetylated-tubulin/α-tubulin, in support of the immunostaining analysis. The relative ratios to the controls were 2.7 ± 0.2 and 1.6 ± 0.1 for tubacin-treatment and α-TAT1-overexpression, respectively (*n* = 3, *p* < 0.01 for both).

GFP-KL1 overexpression was next conducted under conditions of tubacin-treatment and α-TAT1-overexpression. Katanin was included, the enhanced activity of which under tubacin treatment was observed in our previous study [21]. Cells were sorted with regard to the katanin or KL1-expression levels, and a focus was placed on medium expressors, as we did previously [20]. Representative images are shown in Figure 1B with the quantitative data on the microtubule levels indicated in the graph. Medium expressors of katanin showed a 58% loss of microtubules compared with the control, whereas tubacin-enhanced microtubule severing resulted in an 83% loss, as described previously [21]. Consistently, enhanced microtubule severing activity by katanin was also observed in the α-TAT1 expressing cells (82% loss). Medium expressors of KL1 showed a 43% loss compared with the control, also as described previously [20]. A significant upregulation of microtubule severing was found under the various experimental conditions tested (Figure 1B (+KL1 row)). As indicated by the quantification of these results (Figure 1B, graph), the effects of enhanced acetylation were statistically significant. There was a significant increase in microtubule loss by 21% and 18% in tubacin-treated and α-TAT1 overexpressing cells, respectively. Taken together, these results support the hypothesis that microtubules rich in acetylated tubulin are also favored for severing by KL1 in a similar manner to katanin.

### 2.2. KL1 Expression Results in Greater Micronucleation under Enhanced Microtubule Acetylation

RFL-6 rat fibroblasts are immortalized but non-transformed cells. We reported previously that these cells are sensitive to authentic oncogenes in a similar manner to other rodent fibroblast cell lines [20]. In that previous study also, we observed in liquid cultures that anaphase chromosome abnormalities and interphase micronucleation positively correlate with cell transformation activity. In this current study, therefore, the effects of enhanced microtubule acetylation on anaphase and on micronucleation were tested. The effects of tubacin-treatment and α-TAT1-overexpression in stably wild-type KL1-expressing RFL-6 cells were thus analyzed.

**Figure 2:**
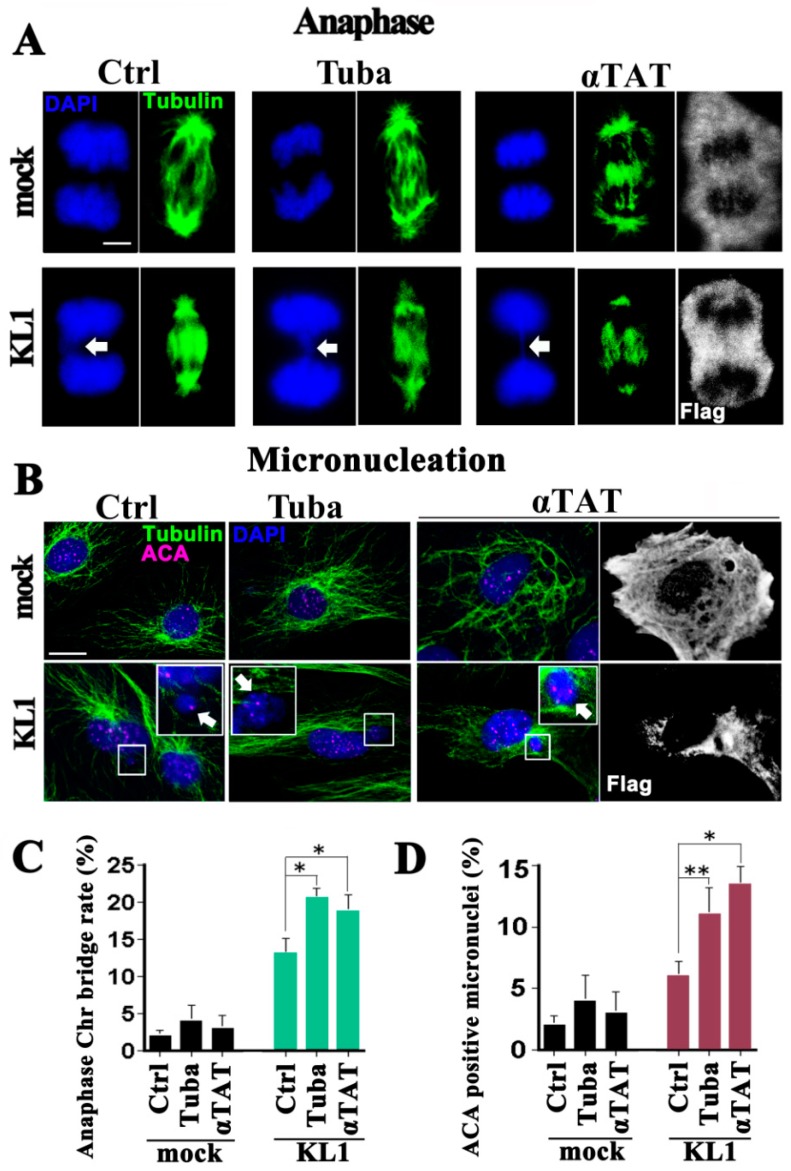
KL1-expressing RFL-6 fibroblasts show elevated chromosome bridging and micronucleation in response to enhanced microtubule acetylation. Equivalent numbers of vector-transfected mock control cells and stably expressing wild-type KL1 cells were plated, treated with tubacin or transfected with α-TAT1 plasmids, cultured and stained with anti-general tubulin (Tubulin), anti-centromere (ACA), or anti-flag (Flag) antibodies and counterstained with DAPI. (**A**) During anaphase, spontaneous chromosome bridges were frequently detected in the KL1-expressing lines. Under microtubule hyperacetylation KL1-expressers but not controls showed increased chromosome bridging (arrows in the DAPI panels). The chromosome bridging morphologies were unchanged either following treatment with tubacin or α-TAT1 overexpression. Bar, 5 µm. (**B**) Cells were stained for tubulin, centromeres (using ACAs), and DNA. In the control cells, ACA signals were detectable in the nucleus. Tubacin treatment (Tuba) or α-TAT1 overexpression (αTAT) did not induce any increase in micronucleation in the control cells. KL1-expressers spontaneously generated micronuclei most of which were ACA-positive (enlarged views; arrows), which were significantly enhanced by treatment with tubacin or by α-TAT1 overexpression. Under these enhancements, there was no change in the number of ACA-positive dots (1–2 dots per micronucleus) or in the number of micronuclei per cell (one per cell on average). Bar, 10 µm. (**C**) Quantification of chromosome bridges. The number of chromosome bridges/number of total anaphases was determined (>100 anaphases were counted). (**D**) Quantification of ACA-positive micronuclei shown in (**B**). KL1-expressing fibroblasts showed significant increases under tubacin treatment or α-TAT1 overexpression (>200 interphase cells were counted). The asterisks and double asterisk indicate significant differences (Student’s *t*-test, *p* < 0.01 and *p* < 0.05, respectively).

Cell proliferation and cell death was first assayed in liquid culture. There was no significant difference found between the untreated and tubacin-treated and α-TAT1-overexpressing cells, either in the case of the controls or KL1-expressers. Regarding the mitotic index, a slight but significant increase in the tubacin-treated and α-TAT1-overexpressing KL1 cells was detected compared with the untreated KL1 cells. Under tubacin-treatment and α-TAT1-overexpression conditions, the mock-transfected control cells showed no abnormalities in their anaphase microtubule morphology nor chromosome separation (Figure 2A,C; mock), and no significant whole chromosome micronucleation changes (Figure 2B,D; mock) as detected by staining for DNA (DAPI) and centromeres (anti-centromere antibody: ACA) [20].

As reported in our previous study, spontaneous anaphase chromosome bridging and micronucleation is evident in KL1-expressing cells (Figure 2A–D) [20]. In the current experiments, tubacin treatment or induced α-TAT1 overexpression in KL1-expressers caused moderate but significant increases in anaphase chromosome bridging (1.7-and 1.5-fold increases for tubacin and α-TAT1, respectively) compared with untreated cells. Similarly, greater micronucleation was observed in both the tubacin-treated and α-TAT1 overexpressing KL1 cells. In the quantitative analysis of these same cells, significant 1.5-and 1.4-fold increases were found in micronucleation for tubacin treatment and α-TAT1 overexpression, respectively.

These data suggested that under conditions of an imbalance between KL1 and microtubule associated proteins (MAPs) including tau [20], the enhanced acetylation of microtubules causes greater abnormalities in chromosome separation and aneuploidy.

### 2.3. Effects of Tubacin and a Tubulin Mutant on Acetylated Microtubules in HMECs

Most human breast cancers originate from normal mammary epithelial cells and we and others have confirmed the expression of endogenous tau in HMECs [20,27,28]. Because we previously observed inhibitory effects of tau on KL1 both in rat fibroblasts and HMECs [20], and because it was found in the present analysis that the enhanced acetylation of microtubules upregulates KL1 activity in rat fibroblasts, it was tested whether the hypothesis of this present study regarding microtubule acetylation could be confirmed in HMECs. The effects of 10 µM tubacin in HMECs were examined under tau knockdown conditions. To selectively cancel out the effects of tubacin on tubulin acetylation, a human α-tubulin mutant was employed, tubulin K40R [4,19], which cannot be acetylated [29].

**Figure 3:**
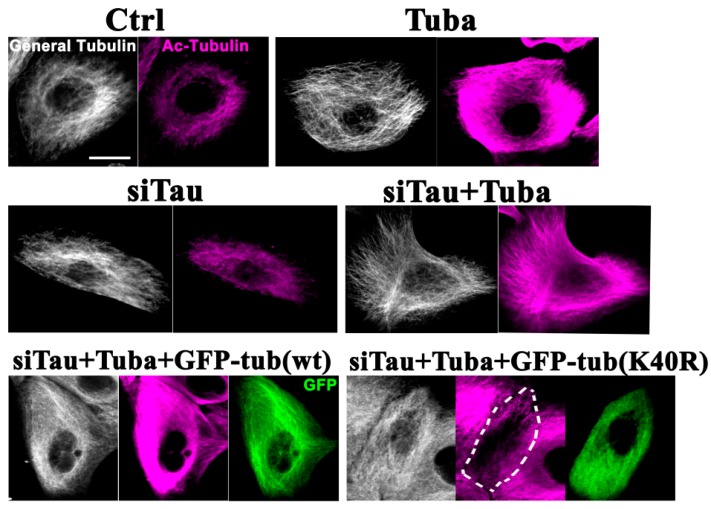
Effects of tubacin treatment and tubulin mutant overexpression on cellular microtubule acetylation in HMECs. HMECs were treated with tubacin and/or anti-tau siRNA with/without transfection of GFP-tagged wild-type α-tubulin or GFP-tagged mutant tubulin (K40R) expression plasmids. The cells were then cultured and stained with anti-tubulin or anti-acetylated tubulin antibodies. White signals denote general tubulin staining (General Tubulin), magenta signals indicate acetylated tubulin (Ac-Tubulin), and green signals indicate GFP. Tubacin-treated cells (Tuba) showed enhanced microtubule acetylation compared with the control cells (Ctrl). Tubacin treatment efficiently upregulated microtubule acetylation under siRNA knockdown of Tau (siTau + Tuba). The microtubule arrays of tubacin-treated cells or anti-tau siRNA-treated cells show no prominent organizational changes compared with the controls. Upon tau knockdown by siRNA, expression of a wild-type tubulin construct did not disrupt the tubacin-mediated enhancement of microtubule acetylation (siTau + Tuba + GFP-tub(wt)) whereas acetylation mutant tubulin expression lowered it to near normal levels (siTau + Tuba + GFP-tub(K40R)). The dotted area denotes a mutant tubulin-expressing cell. Scale bar, 10 µm.

In the interphase HMECs a clear enhancement of microtubule acetylation was observed following exposure to tubacin (Figure 3; Ctrl vs. Tuba). Tau has been shown previously to increase microtubule acetylation [30]. In tau-knockdown HMECs, a slight decrease in microtubule acetylation was detected compared with the control cells. In these same tau knockdown cells, tubacin-treatment produced an efficient enhancement of microtubule acetylation (Figure 3; siTau vs. siTau + Tuba) above the control cells (Figure 3; Ctrl vs. siTau + Tuba). GFP-tagged tubulin (tub) constructs (GFP-tubulin and GFP-tubulin(K40R)) were next expressed in these dual tau-knockdown and tubacin-treated cells. Both of the exogenous proteins expressed from these constructs were successfully incorporated into microtubules (Figure 3; siTau + Tuba + GFP-tub(wt) and siTau + Tuba + GFP-tub(K40R)) as previously reported [31,32]. In the GFP-tubulin expressing cells, microtubules showed hyperacetylation in response to tubacin (Figure 3; siTau + Tuba + GFP-tub(wt)). However, the effects of tubacin were largely canceled out by the exogenous expression of GFP-tubulin(K40R) (Figure 3; siTau + Tuba + GFP-tub(K40R)). Consistent with the immunostaining results, western blot analysis (Figure S1B) revealed a significant increase in the ratios of acetylated-tubulin/α-tubulin in tubacin-treated cells and a non-significant decrease in tau knockdown cells. The relative ratios to the controls were 1.7 ± 0.1 (*p* < 0.01) and 0.8 ± 0.1 for the tubacin-treated and tau-knockdown cells, respectively (*n* = 3, for both). Under dual tau-knockdown and tubacin-treatment conditions, both the control vector and wild-type tubulin expressing cells showed significant increases in the ratios of endogenous acetylated-tubulin/α-tubulin, whereas tubulin(K40R) mutant expression did not, confirming the inhibitory effects of the acetylation mutant. The relative ratios to the controls were 1.5 ± 0.1 (*p* < 0.05), 1.7 ± 0.1 (*p* < 0.01), and 1.1 ± 0.2 for siTau + Tuba, siTau + Tuba + GFP-tub(wt), and siTau + Tuba + GFP-tub(K40R), respectively (*n* = 3, for each).

**Figure 4:**
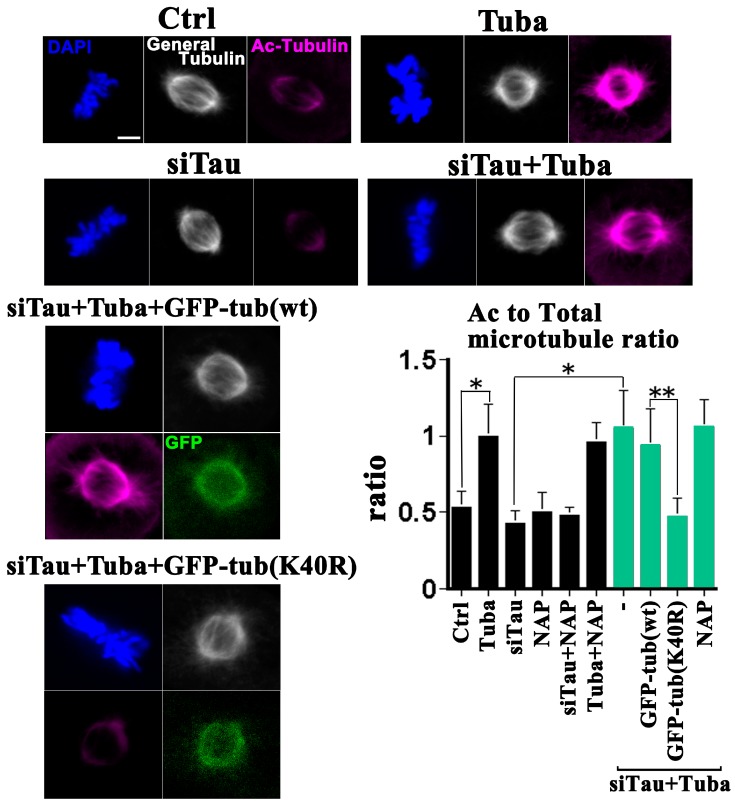
Mitotic effects of microtubule acetylation enhancement in tau knockdown human mammary epithelial cells (HMECs). Metaphase cells were selected for their condensed DNA morphology (detected via DAPI staining) and analyzed for microtubule acetylation. Control cells showed mild acetylation signals between both poles with slight gradients exhibiting a near polar maximum. Compared with the control cells (Ctrl), tubacin-treated cells (Tuba) showed enhanced microtubule acetylation with no abnormal morphologies. The siRNA-treated cells targeting tau (siTau) showed a slight decrease in microtubule acetylation. Under tau knockdown conditions, further tubacin treatment (siTau + Tuba) efficiently increased the microtubule acetylation levels without any morphological changes detected via general tubulin staining. Under both tau siRNA and tubacin treatment (dual treatment), exogenous wild-type tubulin-expressing cells did not show any disruption to the effects of tubacin on spindle microtubule acetylation (siTau + Tuba + GFP-tub(wt)) whereas expression of the acetylation mutant tubulin lowered it to near normal levels (siTau + Tuba + GFP-tub(K40R)). Scale bar, 5 µm. The graph indicates the quantification of acetylated-tubulin/total-tubulin signal ratios in mitotic cells. The total tubulin levels were similar, and no significant differences were detected under any of the experimental conditions. Significant increases in the acetylated-tubulin/total-tubulin signal ratio were detected between the control (Ctrl) and tubacin-treated (Tuba) cells. NAP treatment (NAP) did not produce any change in microtubule acetylation. Tubacin treatment under tau knockdown conditions (siTau + Tuba) produced a similar enhancement to tubacin treatment alone (Tuba). Under the dual conditions, further expression of mutant tubulin but not wild-type reduced the acetylated-tubulin/total-tubulin signal ratio to the control or tau knockdown levels (siTau + Tuba + GFP-tub(wt) vs. siTau + Tuba + GFP-tub(K40R)). The total acetylation levels induced under the dual tubacin treatment and tau knockdown conditions were not significantly changed by further NAP treatments (siTau + Tuba vs. siTau + Tuba + NAP; >25 cells were analyzed). The asterisks and a double asterisk indicate significant differences (Student’s *t*-test, *p* < 0.01 and *p* < 0.05, respectively).

Mitosis was the next focus of this current investigation because we had previously observed that KL1 causes microtubule abnormalities at the mitotic spindle in HMECs [20]. In prometa/metaphase cells, efficient enhancement of mitotic spindle microtubule acetylation was observed following tubacin treatment (Figure 4; Ctrl vs. Tuba). A slight tendency toward a decreased microtubule acetylation in the tau-knockdown cells was noted (Figure 4; Ctrl vs. siTau) but further tubacin treatment produced an efficient enhancement of acetylation (Figure 4; siTau vs. siTau + Tuba). This enhancement was inhibited in the cells expressing the acetylation mutant tubulin (Figure 4; siTau + Tuba vs. siTau + Tuba + GFP-tub(K40R)), whereas no such inhibition was observed with the wild-type tubulin (Figure 4; siTau + Tuba vs. siTau + Tuba + GFP-tub(wt)). Quantitation analysis indicated a significant 1.8-fold increase in the ratio of acetylated-tubulin to total-tubulin under tubacin treatment (Figure 4, graph; Ctrl vs. Tuba). There was also a trend toward a near 10% decrease in this ratio in tau-knockdown cells, but this was not a significant reduction (Figure 4 graph; Ctrl vs. siTau). The tubacin-treated tau-knockdown cells showed a similar level of microtubule acetylation to that of the tubacin-treated control cells (Figure 4 graph; Tuba vs. siTau + Tuba). Exogenous wild-type tubulin overexpression produced a comparable level of enhanced microtubule acetylation to that of the tau knockdown cells treated with tubacin. However, the mutant tubulin (K40R) construct-expressing cells showed clear inhibition of the effects of tubacin (Figure 4 graph; siTau + Tuba + GFP-tub(wt) vs. siTau + Tuba + GFP-tub(K40R)). In these mutant-expressing cells, the level of microtubule acetylation also appeared to return to that of the controls or siTau alone cells (Figure 4 graph; Ctrl vs. siTau + Tuba + GFP-tub(K40R)). Wild-type tubulin overexpression in the control or tau-knockdown HMECs induced no changes in microtubule acetylation compared with no overexpression, consistent with the findings of a previous report [31] and did not disrupt the microtubule acetylation-enhancing effects of tubacin. Mutant tubulin overexpression in control or tau-knockdown cells reduced the microtubule acetylation to below control levels, as also described previously [19]. The tau knockdown alone, mutant K40R tubulin overexpression under tubacin treatment alone, and mutant K40R tubulin overexpression under these dual conditions produced similar microtubule acetylation levels.

Based on these findings, the effects of microtubule hyperacetylation on aneuploidization-related phenomena in HMECs were explored.

### 2.4. Tau Knockdown HMECs Show Increased Chromosome-Bridging and Micronucleation in Response to Enhanced Microtubule Acetylation

Katanin has been shown to be enhanced by microtubule acetylation in our previous reports and we also recognized in these prior studies that tau masks the effects of microtubule acetylation on katanin [21,26]. In addition, we have demonstrated previously that tau has inhibitory effects on both katanin and KL1 [20,26]. Based on the similarities we observed in the regulation of KL1 and katanin, it was reasonable to speculate that similar relationships would exist between KL1 and katanin in tau and microtubule acetylation. In our previous study in tau knockdown HMECs, we observed that micronucleation was dependent on KL1 [20]. To further assess that biological outcome of microtubule hyperacetylation in the present experiments, the acetylation levels in HMECs were enhanced by tubacin treatment under a tau knockdown background.

**Figure 5:**
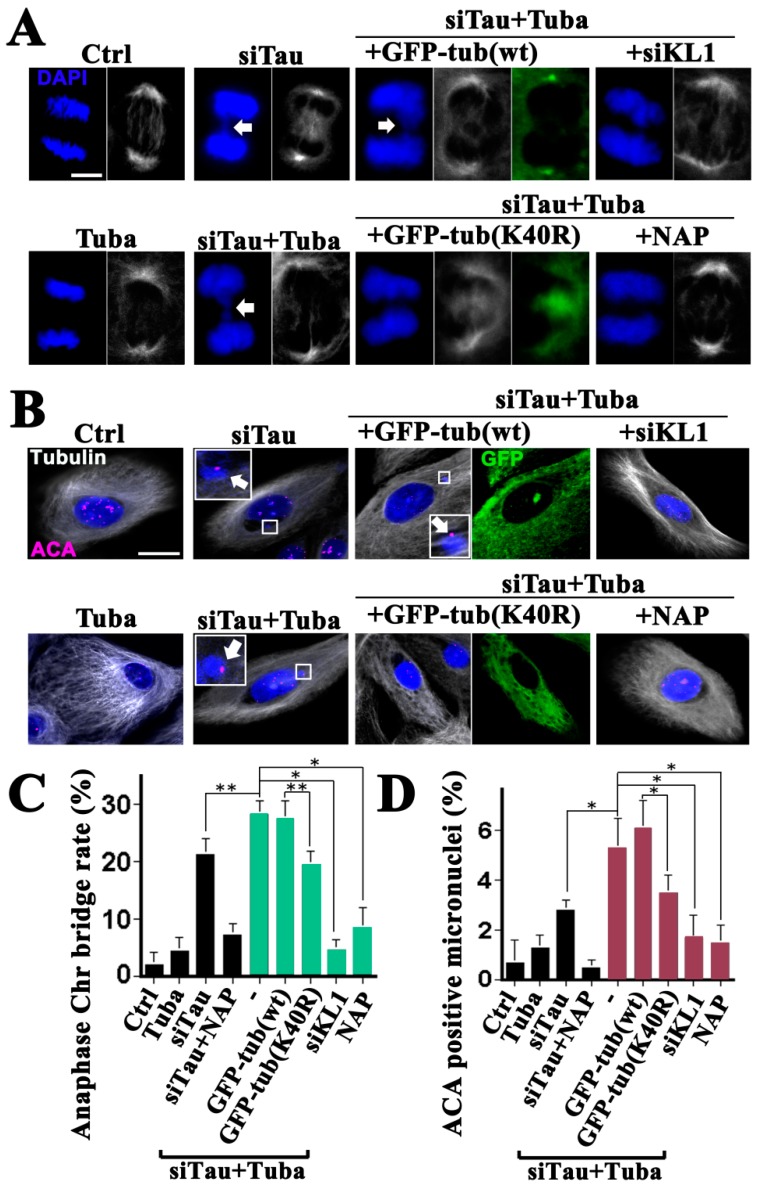
Tubacin treatment exacerbates tau knockdown-induced chromosome bridging and micronucleation in HMECs. HMECs were treated with tubacin (Tuba) and/or 30 nM NAP, transfected with siRNA targeting tau (siTau) with/without siRNA for KL1 (siKL1), and transfected with wild-type tubulin (GFP-tub(wt)) or unacetylatable tubulin mutant (GFP-tub(K40R)) constructs. The cells were then stained for general tubulin (Tubulin), centromeres (ACA), and DNA (DAPI). (**A**) Anaphase chromosome bridging analysis. Arrows indicate chromosome bridging. Chromosome bridging in tau-knockdown cells (siTau) was enhanced by tubacin treatment (siTau + Tuba: dual treatment). Treatment with tubacin alone did not induce chromosome bridging compared with the control cells. The dual tubacin treatment and tau knockdown-induced enhanced bridging was largely inhibited by a further KL1 knockdown (siTau + Tuba + siKL1). Under tubacin treatment and tau knockdown conditions, overexpression of wild-type tubulin did not affect enhanced bridging (siTau + Tuba + GFP-tub(wt)) whereas acetylation mutant tubulin expression inhibited this (siTau + Tuba + GFP-tub(K40R)). Further NAP treatment also recovered the normal anaphase chromosome morphology of the tubacin-treated cells under tau knockdown conditions (siTau + Tuba + NAP). Bar, 5 µm. (**B**) Interphase HMECs were assessed for ACA-positive micronucleation. Tubacin treatment alone did not induce micronucleation (Tuba). Compared with a tau knockdown by siRNA (siTau), further tubacin treatment increased the micronucleation levels (siTau + Tuba). Under the dual tubacin treatment and tau knockdown conditions, a further knockdown of KL1 in HMECs inhibited micronucleation (siTau + Tuba + siKL1). Furthermore, in these tubacin-treated tau knockdown cells, overexpression of the acetylation mutant tubulin suppressed micronucleation (siTau + Tuba + GFP-tub(K40R)) unlike exogenous wild-type tubulin (siTau + Tuba + GFP-tub(wt)). NAP treatment also reduced the micronucleation levels (siTau + Tuba + NAP). The enlarged views show ACA-positive micronuclei (arrows). Bar, 10 µm. (**C**) Bar graph showing quantification results for anaphase chromosome bridges in HMECs (>40 cells were counted). (**D**) Bar graph showing quantification results for ACA-positive micronucleation in HMECs (>200 cells were counted). The asterisks and double asterisks indicate significant differences (Student’s *t*-test, *p* < 0.01 and *p* < 0.05, respectively).

The tubacin-treated HMECs showed no significant proliferation or mitotic index changes, and no obvious alterations in their mitotic spindle morphologies, compared with the controls (Figure 4; general tubulin images of Ctrl vs. Tuba, Figure 5A,C; Ctrl vs. Tuba). There was also no observed increase in micronucleation (Figure 5B,D; Ctrl vs. Tuba). Under tau knockdown conditions however, a significant increase in anaphase chromosome bridging and micronucleation was detected (Figure 5A–D; Ctrl vs. siTau) as reported in our previous study [20]. Under tau knockdown and tubacin treatment conditions, increased anaphase chromosome bridging was evident (Figure 5A; siTau vs. siTau + Tuba). However, there was no increase in morphological abnormalities. The increase (about 1.3-fold) in the rate of anaphase chromosome bridging was moderate but significant (Figure 5C; siTau vs. siTau + Tuba). Consistent with these anaphase data, a significant 1.8-fold increase in micronucleation was observed under the same conditions (Figure 5B,D; siTau and siTau + Tuba). Based on these current findings and previously reported results [20], a further knockdown of KL1 was done in tau knockdown cells which were then treated with tubacin. Following this KL1 knockdown, chromosome bridging and micronucleation returned to basal levels (Figure 5A–D, Ctrl vs. siTau + Tuba + siKL1), suggesting that the major response factor to hyperacetylation in the observed enhanced phenotypes may be endogenous KL1.

Even though tubacin is a domain2 specific inhibitor of HDAC6 [33] and would therefore be expected to have less impact on its histone deacetylase activity, the possibility of non-tubulin acetylation-dependent effects could not be excluded. To determine whether the elevation in KL1-dependent micronucleation was really due to enhanced microtubule acetylation, the effects of the tubulin acetylation mutant were further tested. Wild-type tubulin overexpression did not influence the rates of chromosome bridging either under dual tau knockdown and tubacin treatment conditions (Figure 5A,C; siTau + Tuba vs. siTau + Tuba + GFP-tub(wt)) or under tau knockdown alone. Remarkably however, the K40R mutant-expressing cells showed reduced chromosome bridging to the same levels as the tau knockdown cells (Figure 5C; siTau vs. siTau + Tuba + GFP-tub(K40R)). Expression of this mutant caused a reduction in microtubule acetylation to control levels in the presence of tubacin (Figure 4). It was speculated therefore that the mutant effects were caused by blocking tubacin rather than other possible mechanisms of decreased microtubule acetylation. In accordance with the reduced level of abnormalities during anaphase, the K40R mutant tubulin expressing cells showed similar levels of micronucleation under dual tubacin treatment and tau knockdown conditions to those seen in the tau knockdown alone cells (Figure 5B,D; siTau vs. siTau + Tuba + GFP-tub(K40R)).

These data supported the notion that the elevated micronucleation was caused by enhanced microtubule acetylation by tubacin.

In our previous study, we found that a membrane permeable octapeptide, NAP, (or Davunetide) efficiently compensated for a reduction in tau function and protected neuronal axons from the microtubule reduction caused by katanin [26]. We found in that study that decreased microtubule acetylation by HDAC6 overexpression also prevented the axonal loss of microtubules caused by katanin. However, these effects at the axons were seen only under tau knockdown conditions. With these backgrounds in mind, the issue of whether NAP has preventive effects on micronucleation under conditions of enhanced microtubule acetylation was assessed in this present study.

Whether NAP affects the acetylation of microtubules was first evaluated in this series of analyses. NAP-treated cells (30 nM) showed no change in microtubule acetylation during interphase and mitosis (Figure 4, graph; Ctrl vs. NAP). Under tau knockdown conditions however, NAP-treated cells showed a slight increase compared with tau knockdown alone during interphase. Furthermore, NAP treatment did not inhibit the effects of tubacin on microtubule acetylation either in interphase or mitosis (Figure 4, graph; Tuba vs. Tuba + NAP). These data suggested that NAP is not a potent modifier of microtubule acetylation.

NAP treatment of HMECs significantly inhibited both chromosome bridging and micronucleation under a tau knockdown background, as reported previously (Figure 5C,D; siTau vs. siTau + NAP) [20]. Interestingly, significantly higher than 70% reduction in both chromosome bridging and micronucleation was evident in the NAP-treated cells under tau knockdown and tubacin treatment conditions (Figure 5A–D; siTau + Tuba vs. siTau + Tuba + NAP). These data indicated that NAP inhibits enhanced KL1 activity caused by microtubule acetylation not by directly suppressing acetylation but via other mechanisms.

## 3. Discussion

In the present study, the severing activity of KL1 was analyzed under conditions of enhanced microtubule acetylation. Together with our previous findings [20], the current study results revealed that KL1 has hyperactive effects when not balanced by tau in both fibroblasts and HMECs and that excessive KL1 activity-induced chromosome bridging and micronucleation are further enhanced by elevated microtubule acetylation in both cell types. These data suggest that the enhanced microtubule acetylation observed in breast cancer patients [19] also induces KL1-dependent aneuploidization through mitotic abnormalities in early breast carcinogenesis when tau is deficient.

Three proteins have been reported to be involved in tumorigenic processes that occur via altered spindle microtubule acetylation. Kindlin1 is a causal gene for Kindler Syndrome which results in a predisposition to squamous cell carcinoma [15]. Loss of Kindlin1 leads to lowered microtubule acetylation via a loss of an inhibitory interaction with HDAC6 and causes abnormalities in asymmetric division in skin [15]. RBP-J interacting and tubulin-associated protein (RITA) is downregulated in hepatocellular carcinoma [16]. During mitosis, RITA facilitates HDAC6 interactions with spindle microtubules. The loss of RITA has been shown to cause hyperacetylation of microtubules and chromosome segregation errors [16]. Brain-expressed X-linked member 4 (BEX4) is overexpressed in tumors and is linked to aneuploidy [34]. BEX4 binds and inhibits Sirt2, causing the hyperacetylation of spindle microtubules [34]. In this regard, it is noteworthy that tau has been shown to interact with and inhibit HDAC6 [35].

How, though, could a modification of α-tubulin that occurs on the luminal face of the polymer alter the sensitivity of the microtubule to KL1? A possibility is that a severing protein domain reaches into the microtubules through the lattice and pulls out a subunit when causing the microtubule to break, as described by us previously [21]. In that process the luminally localized acetylation of K40 could be recognized by that domain, resulting in a severing increase through an enhanced affinity for the microtubule surface or through augmented mechanochemical coupling which occurs inside the hexamer [36].

A question that arises from the existing evidence is why a hierarchy appears to exist in the regulation of KL1, that is, why the enhanced effects of microtubule acetylation are observed only under tau knockdown conditions. Microtubule acetylation has been shown previously to constrain protofilament numbers and maintain uniformity of the luminal diameter of microtubules [29,37]. Some proteins are considered to be transported inside the lumen [38,39]. Two separate functions of microtubule acetylation may therefore exist i.e., to prompt the efficient transportation of luminal proteins and, simultaneously, to sensitize microtubules to certain severing proteins. In this regard, the protection of acetylated microtubules from severing by tau might have functional relevance when the former function of microtubule acetylation has priority because a mechanism that promotes the transportation of luminal materials, and at the same time induces the rupture of the tubules needed for that process, would not be able to function properly. A previous report has indicated that the tau protein itself also has constraining effects on the number of microtubule protofilaments at relatively low molar ratios to tubulin and suggested a possible “allosteric role” of tau [40]. Tau might therefore facilitate luminal transportation together with microtubule acetylation. The relatively low molar ratio of tau to tubulin observed at the mitotic spindle [20] also supports this idea.

The preventive effects of NAP against microtubule acetylation-induced KL1 activity without influencing microtubule acetylation are somewhat of a mystery at present. One possibility might be that NAP exerts its effects through a decrease of toxic tau species. However, significant elevations in chromosome bridging and micronucleation were observed only under tau knockdown conditions, which reduces the tau expression levels to about 10% of the controls [20]. It is thus possible that the observed effects of acetylation in the current study experiments are not through increase of toxic tau species. In support of this idea, both in chromosome bridging and micronucleation, the inhibiting effects of salicylate, which has been reported to reduce toxic tau species via the downregulation of tau acetylation at K174 [41], were not evident (Figure S2). Recent research has suggested that NAP can recruit a limited amount of tau to the microtubules [42] even under tau knockdown conditions. Regardless of the mechanisms of action, the current study findings suggests that NAP suppresses aneuploidization, which is a possible downstream event following microtubule hyperacetylation.

The direct control of microtubule acetylation may be therapeutically attractive [26]. The present study data suggest that tau-deficient hyperacetylated breast primary tumor cells could be an appropriate target for novel anti-cancer agents. However, in any such strategy, attention should also be paid to the normal microtubule acetylation levels because a decrease below these levels has also been suggested to cause abnormal mitosis [15]. The finely-tuned regulation of microtubule acetylation might be crucial for a normal functioning mitotic spindle in some cell types [13,34]. Tumor biology considerations aside, it must also be noted that decreased microtubule acetylation has been demonstrated to cause Gulf War illness in which tubacin has been considered as a candidate drug [43]. The inhibition of fidgetin, one of the microtubule-severing proteins, might be another potential approach to decreasing the microtubule acetylation ratio because fidgetin preferably severs unacetylated microtubules [44,45]. Such an approach could be expected to work when microtubule acetylation is only moderately enhanced.

The present study findings are potentially relevant to cancer in another way, particularly in cases that are also suffering from diabetes mellitus. It has been shown that patients with type 2 diabetes have an increased risk of developing breast cancer [46], that high glucose induces microtubule hyperacetylation in cultured schwannoma cells [47], and that hyperacetylation of microtubules, possibly through the inhibition of Sirt2, occurs in the tissues of an animal model of diabetes [48]. In diabetes, peripheral cells do not efficiently uptake glucose from the extracellular fluid due to the reduced insulin function. To prevent cellular starvation, ketone bodies (primarily β-hydroxybutyrate and acetoacetic acid) are likely to be overproduced in the liver. These two ketone bodies enter the peripheral cells where they are converted to acetyl-CoA to be used for energy. As described earlier, acetyl-CoA is also a substrate for α-TAT1.

## 4. Materials and Methods

### 4.1. Antibodies and Reagents

A rabbit polyclonal antibody against DDDDK-tag was used as the anti-Flag antibody (Medical and Biological Laboratories Co., Ltd., Nagoya, Japan). Mouse monoclonal antibodies against α-tubulin (DM1A (epitope; 426–450 residues), NeoMarkers, Fremont, CA, USA), Cy3-conjugated-β-tubulin (both antibodies used for general tubulin staining), and acetylated-α-tubulin (clone 6-11B-1) (Sigma, St. Louis, MO, USA), were used. Human anti-centromere antibody (Antibodies Inc., Davis, CA, USA) was also used. DAPI (4,6-diamidino-2-phenylindole dihydrochloride hydrate) (Sigma), and tubacin (Enzo Life Sciences, Farmingdale, NY, USA) were used. Salicylate (sodium salicylate) was purchased from Sigma. NAP peptide (Biorbit, San Francisco, CA, USA) was aliquoted and stocked as a 1 mM solution in 75% dimethyl sulfoxide at −20 °C.

### 4.2. Expression Constructs

Human KL1-expression vector was sourced commercially (RC220944; OriGene Technologies, Rockville, MD, USA) and the KL1 protein was expressed in a pEGFP-C1 vector (Clontech, Mountain View, CA, USA) (GFP-KL1) as previously reported [20]. Mouse α-TAT1 expression vector was also purchased commercially (MR206707; OriGene Technologies) and flag-tagged α-TAT1 was expressed from a pCMV6-entry vector. The expression vector for rat katanin (pEGFP-C1-p60 katanin) was kind gift of Dr. PW. Baas, Drexel University, Philadelphia, PA. The human α-tubulin construct [20] was N-terminally GFP-tagged and expressed in pEGFP-C1 (GFP-tubulin(wt)). The tubulin K40R mutation was introduced using a GeneArt Site-Directed Mutagenesis PLUS kit (Life Technologies, Carlsbad, CA, USA) (GFP-tubulin(K40R)) and the structure of the construct was confirmed by sequencing.

### 4.3. Cell Culture, Transfection and Drug Treatments

Animal fibroblasts. Rat RFL-6 fibroblasts were purchased from the Health Protection Agency (Porton Down, Salisbury, UK), cultured as described previously [20], and transfected with expression plasmids using the electroporation device NEPA GENE CUY21Pro-Vitro (Ichikawa, Chiba, Japan) and employing previously reported settings [20]. The transfection efficiency was about 30–40%. The protocols used for electroporation, plating, and cell culture were as described previously [20]. For chromosome-bridging detection and micronuclei detection, cells were transfected with α-TAT1 expression plasmid or treated with tubacin (10 µM). After two days of culture, the cells were fixed and subjected to immunostaining or western blotting.

Human mammary epithelial cells. Human (female) mammary epithelial cells (HMEC, CC-2551; Lonza, Basel, Switzerland) were purchased at passage 7, PD 18 and cultured as described previously [20]. Transfection of HMECs with GFP-tubulin or GFP-tubulin (K40R) expression plasmids with or without anti-tau siRNA and/or tubacin treatments was performed using a TransIT-X2 Dynamic Delivery System (Mirus, Madison, WI, USA). Salicylate was used at 5 mM as described previously [41]. For GFP-tubulin construct overexpression, cells were plated on 35 mm culture dishes and 2.5 μg of the plasmid was transfected. The transfection efficiency was about 10–20%. KL1 and/or tau knockdown were performed as previously described [20].

For NAP treatment, cells were transfected with siRNAs, with or without tubacin (10 µM) treatment, and simultaneously treated with NAP (30 nM). Chromosome-bridging detection and micronuclei detection were performed after two days of culture.

### 4.4. Immunofluorescence Techniques

The immunostaining experiments were performed as described previously [20]. Briefly, for centromere staining with ACA, cells were fixed with ice cold methanol + acetone (1:1) for 15 min. For staining of acetylated tubulin and general tubulin, cells were fixed and extracted with 2% paraformaldehyde, 0.1% glutaraldehyde, and 0.2% triton X100 in PHEM [20] for 30 min at room temperature. Fluorescence signals were detected using a confocal laser scanning microscope, LSM 700 (Carl Zeiss, Thornwood, NY, USA) using a Plan-Apochromat 40 (oil) objective lens with a 1.3 aperture. The original magnification was ×400. To quantify the microtubule levels, cells were simultaneously fixed and extracted to remove free tubulin and then immunostained as described previously [20]. Images to be compared were taken at identical settings in terms of exposure time, brightness and contrast and analyzed with ZEN 2012 software (Carl Zeiss). For the microtubule sensitivity test, medium expressers were chosen as described previously [20]. Measures of the total microtubule levels or acetylated microtubule levels were taken as the total fluorescence intensity per cell using the analytical command for the intensity mean value in the ZEN software. Values were expressed as arbitrary fluorescent units (AFUs). Three independent experiments were performed in each study. Data represent the mean ± SD. Statistical analyses were done using the Student’s *t*-test.

### 4.5. Western Blot Analysis

Western blotting and subsequent quantification of the signals was performed as described previously [20]. For the quantitation of acetylated-tubulin whole cell lysates of RFL-6 cells or HMECs were analyzed based on the previous report [49].

## 5. Conclusions

Microtubule hyperacetylation is associated with a poorer prognosis in breast cancer. KL1 is activated in tau-deficient breast cancer cells and is a potential aneuploidy generator. The present study data suggest the possibility that under microtubule hyperacetylation, KL1 causes more aneuploidization and further promotes breast tumorigenesis.

## Supplementary Materials

The following are available online at http://www.mdpi.com/1422-0067/19/9/2488/s1.

## Abbreviations

KL1	katanin-like1
MAP	microtubule associated protein
HDAC	histone deacetylase
TAT	tubulin *N*-acetyltransferase
RFL	rat fibroblast
GFP	green fluorescent protein
DAPI	4,6-diamidino-2-phenylindole dihydrochloride hydrate
ACA	human anti-centromere antibody
HMEC	human mammary epithelial cell
Tuba	tubacin
tub	tubulin
NAP	activity-dependent neuroprotective protein (ADNP)-derived octapeptide: NAPVSIPQ
RITA	RBP-J interacting and tubulin-associated protein
BEX4	brain-expressed X-linked member 4
flag	FLAG-tag: polypeptide (DYKDDDDK) protein tag
SS	sodium salicylate
Sirt	sirtuin
