# Supplementary material for: Microtubule Hyperacetylation Enhances KL1-Dependent Micronucleation under a Tau Deficiency in Mammary Epithelial Cells
Source: Int J Mol Sci. 2018 Aug 23;19(9):2488. doi: 10.3390/ijms19092488 (PMC6165458; doi:10.3390/ijms19092488)
Supplement: Supplementary file 1 [file ijms-19-02488-s001.pdf]

Supplementary Materials: Microtubule Hyperacetylation Enhances KL1-Dependent Micronucleation under a Tau Deficiency in Mammary Epithelial Cells

Haruka Sudo

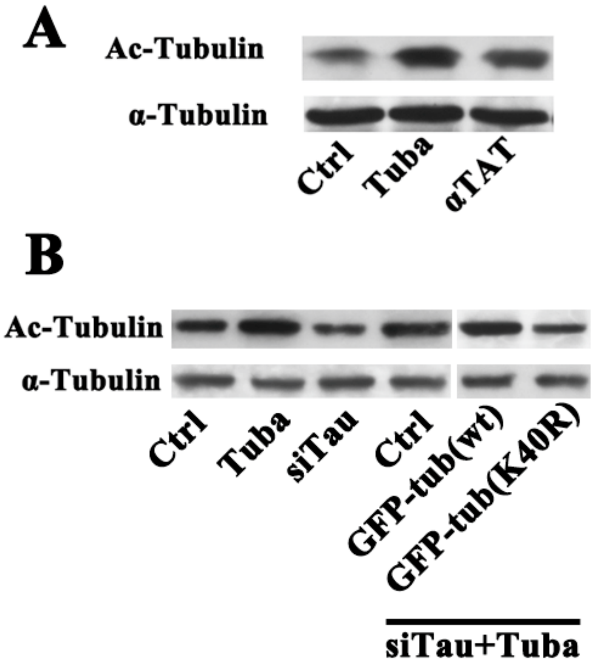

Figure S1. Western blot analysis of the effects of different experimental manipulations on endogenous microtubule acetylation. (**A**) RFL-6 cells were treated with tubacin (Tuba) or transfected with α-TAT1 expression plasmids (αTAT), and whole cell lysates were subjected to immunoblotting. (**B**) HMECs were treated with tubacin (Tuba), transfected with siRNA targeting tau (siTau), and transfected with wild-type tubulin (GFP-tub(wt)) or unacetylatable tubulin mutant (GFP-tub(K40R)) constructs. Whole cell lysates were then subjected to immunoblotting.

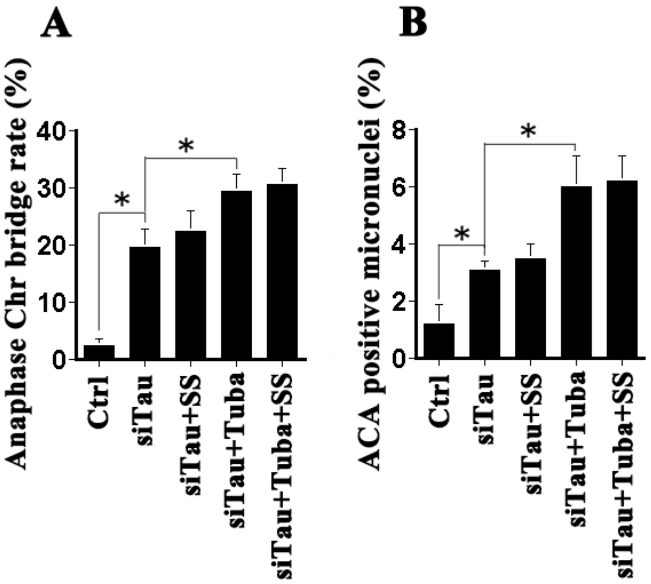

Figure S2. Effects of salicylate (SS) on tubacin-induced micronucleation in HMECs. HMECs were treated with tubacin (Tuba) and/or 5 mM SS, transfected with siRNA targeting tau (siTau). The cells were then stained for general tubulin, centromeres, and DNA as described also in Figure 5. (**A**) Bar graph showing quantification results for anaphase chromosome bridges in HMECs (>40 cells were counted). (**B**) Bar graph showing quantification results for ACA-positive micronucleation in HMECs (>200 cells were counted). No significant effects were observed on chromosome bridging or micronucleation. The asterisks indicate significant differences (Student's *t*-test, $p < 0.01$).
